# RT-PCR Detection of SARS-CoV-2 among Individuals from the Upper Silesian Region—Analysis of 108,516 Tests

**DOI:** 10.3390/diagnostics12010007

**Published:** 2021-12-21

**Authors:** Adam Konka, Mateusz Lejawa, Jadwiga Gaździcka, Aneta Bochenek, Martyna Fronczek, Joanna Katarzyna Strzelczyk

**Affiliations:** 1 Silesian Park of Medical Technology Kardio-Med Silesia, M. Curie-Skłodowskiej 10C, 41-800 Zabrze, Poland; mateusz.lejawa@gmail.com (M.L.); a.bochenek@kmptm.pl (A.B.); m.fronczek@kmptm.pl (M.F.); 2 Department of Pharmacology, Faculty of Medical Sciences in Zabrze, Medical University of Silesia in Katowice, Jordana 38 Str., 41-808 Zabrze, Poland; 3 Department of Medical and Molecular Biology, Faculty of Medical Sciences in Zabrze, Medical University of Silesia, Jordana 19 Str., 41-808 Zabrze, Poland; jgazdzicka@sum.edu.pl (J.G.); jstrzelczyk@sum.edu.pl (J.K.S.)

**Keywords:** SARS-CoV-2, COVID-19, RT-PCR, SARS-CoV-2 variants

## Abstract

Background: The COVID-19 pandemic triggered by the novel severe acute respiratory syndrome coronavirus 2 (SARS-CoV-2) has left a huge mark on everyday lives, introducing restrictions and plunging the global economy. This study aimed to analyze the available epidemiological data from the register of one of the largest laboratories testing for SARS-CoV-2 in the Silesian voivodship of Poland. Methods: This analysis is based upon the epidemiological records collected between 30 March 2020, and 30 April 2021, by the Silesian Park of Medical Technology Kardio-Med Silesia (Zabrze, Poland). In addition, we performed SARS-CoV-2 variant detection in samples from patients reinfected with SARS-CoV-2. Results: Our results confirm that SARS-CoV-2 infections are more common in urban areas. Laboratory-confirmed COVID-19 cases represent 13.21% of all RT-PCR test results during the 13 months of our laboratory diagnostics for SARS-CoV-2 infections. Detection of SARS-CoV-2 variants in samples of potentially reinfected patients showed discrepancies in the results. Conclusions: Due to the higher risk of SARS-CoV-2 infection among the Upper Silesian population, the region is at greater risk of deteriorating economic situation and healthcare as compared to other areas of Poland. RT-PCR methods are inexpensive and suitable for large-scale screening, but they can be untrustworthy so detection of SARS-CoV-2 variants in samples should be confirmed by sequencing.

## 1. Introduction

In December 2019, a novel coronavirus was identified in patients hospitalized for pneumonia in Wuhan, Hubei Province in China. Previously known as 2019 novel coronavirus (2019-nCoV), the isolated virus was classified into beta coronaviruses and is the seventh member of coronaviruses infecting humans [1]. Based on taxonomy and phylogenetic analysis of 2019-nCoV, the virus was renamed as severe acute respiratory syndrome coronavirus 2 (SARS-CoV-2) [2], which causes the coronavirus disease named COVID-19 [3]. The common symptoms of COVID-19 are fever, cough, myalgia or fatigue, dyspnea, shortness of breath as well as less common manifestations, such as sore throat, headache, and diarrhea [4,5,6,7]; however, some infections of SARS-CoV-2 are asymptomatic [8]. Severe cases require hospitalization and intensive care with non-invasive or invasive ventilation and extra-corporeal membrane oxygenation [6,7]. According to recommendations issued by the World Health Organization (WHO), the real-time reverse-transcription polymerase chain reaction (RT-PCR) is one of the tests used to confirm the incidence of COVID-19 [9]. By 2 May 2021, i.e., since the beginning of the pandemic, WHO had reported more than 151 million confirmed infected cases of COVID-19 and 3 million deaths worldwide [10]. In Poland, the first case of COVID-19 was confirmed on 4 March 2020 [11]. Since then and before 4 May 2021, Poland confirmed 2,761,893 cases of infection with SARS-CoV-2, while 352,469 were reported in Upper Silesia [12], which has a population density as high as 366 people per 1 km^2^ [13]. Currently, there are 30 laboratories throughout the Upper Silesian region authorized by the Ministry of Health to carry out COVID-19 testing [14].

The aim of this study was to analyze the statistical incidence of COVID-19 in Silesia, based on real-time RT-PCR tests performed during 13 months from the beginning of the pandemic (March 2020–April 2021) by one of the laboratories in the Silesian agglomeration, from the beginning of the pandemic.

## 2. Materials and Methods

### 2.1. Patients and Specimens

This analysis was based upon medical laboratory records taken by the Silesian Park of Medical Technology Kardio-Med Silesia (Zabrze, Poland) between 30 March 2020 and 30 April 2021. The data included results of SARS-CoV-2 RT-PCR tests and information on age, sex and place of residence. All the data were anonymized. Moreover, the remnant RT-PCR samples (nasopharyngeal swab or nasal and throat swab) confirming COVID-19 were collected. The samples were patient swabs obtained by the laboratory during the standard diagnostic procedure for SARS-CoV-2 detection. Excess buffer from the transferred swab was pipetted into an eppendorf tube and preserved long-term at 80 °C for research purposes. Following approval by the Bioethics Committee and after respective funding was granted, the samples were used to perform the analyses described.

### 2.2. RNA Isolation

The first step to detect SARS-CoV-2 virus was isolation of SARS-CoV-2 nucleic acids from the nasopharyngeal swab or nasal and throat swab, using the isolation kits EliGene Viral DNA/RNA Isolation Kits (Elisabeth Pharmacon, Brno-Zidenice, Czech Republic); FastPure Viral DNA/RNA Mini Kit (Vazyme Biotech, Nanjing, China); Maxwell RSC Viral Total Nucleic Acid Purification Kit (Promega, Madison, WI, USA); Bosphore Viral RNA Extraction Spin Kit (Anatolia Geneworks, Istanbul, Turkey); Nucleospin Dx Virus (Macherey-Nagel, Düren, Germany); Syngen Viral MiniKit PLUS (Syngen, Wroclaw, Poland); Viral Nucleic Acid Extraction Kit Magcore (RBC Bioscience, New Taipei City, Taiwan); Viral Nucleic Acid Isolation Kit (Roche, Basel, Switzerland); TANBead Nucleic Acid Extraction Kit (TANBead, Taoyuan City, Taiwan); Virus DNA/RNA Purification Kit (Biocomma, Guangdong, China).

### 2.3. RT-PCR Assays

The presence of SARS-CoV-2 virus was detected using mainly the tests COVID-19 Real Time Multiplex RT-PCR Kit (Labsystems Diagnostics Oy, Vantaa, Finland) or SARS-CoV-2 Real Time PCR LAB-KIT (BioMaxima S.A., Lublin, Poland). In addition, we made use of the following tests: Bosphore Novel Coronavirus (2019-nCOV) Detection Kit (Anatolia Geneworks, Istanbul, Turkey); DiaPlexQ™ Novel Coronavirus (2019-nCoV) Detection Kit (SolGent Co., Ltd., Daejeon, Korea); Liferiver Novel Coronavirus (2019-nCoV) Real Time Multiplex RT-PCR Kit (Shanghai ZJ Bio-Tech Co., Ltd., Shanghai, China); LightMix Modular SARS-CoV-2 (Covid19) RdRP, E-gene (Roche, Basel, Switzerland); Novel Coronavirus (2019-nCoV) Nucleic Acid Diagnostic Kit (Sansure Biotech, Changsha, China); or VIASURE SARS-CoV-2 Real Time PCR Detection Kit (Certest Biotec S.L., Saragossa, Spain). To perform RT-PCR tests, the following real time PCR instrument were used: AriaMx Real-Time PCR System (Agilent Technologies, Santa Clara, CA, USA), LineGene 9600 Plus (Bioer Technology, Hangzhou, China), Cobas Z480 (Roche, Basel, Switzerland), or LightCycler 480II (Roche, Basel, Switzerland). Information regarding interpretation of the CT values for the passive and non-conclusive results used in standard laboratory tests is presented in the Supplement in Table S1. The inconclusive results were defined on the basis of recommendation of the Polish National Institute of Hygiene in the field of SARS-CoV-2 molecular diagnostics.

### 2.4. Identification of SARS-CoV-2 Variants

To differentiate between the SARS-CoV-2 variants, lineages B.1.1.7 (United Kingdom, UK), B.1.351 (South Africa, ZA), and P.1 (Brasilia, BRA) two assays were used: GeneProof SARS-CoV-2 Vaccine Escape PCR Kit (GeneProof a.s., Brno, Czech Republic) and Virella SARS-CoV-2 Mutant Real-Time RT-PCR Kit (Gerbion GmbH & Co. KG, Kornwestheim, Germany). The latter kit further differentiates the variant SARS-CoV-2-RNA Lineage B.1.258/Cluster V (Mink, DK). Suitable for testing with this kit are samples with a CT value < 32. According to the manufacturers’ instructions of the kit, the CT for del69/70, del241–243 and H655Y mutations should be within the range 19–22, while CT for detecting the N501Y mutation should be 22–25. In the GeneProof SARS-CoV-2 Vaccine Escape PCR Kit the threshold value for detecting the E484K mutation must be set at 15% of the fluorescence value of the positive control. Both kits used had in vitro diagnostic certification for Europe (CE IVD). The reactions were performed with the use of a LineGene 9600 Plus thermocycler (Bioer Technology, Hangzhou, China).

## 3. Results

A total of 108,516 tests were performed during the period of one year when SARS-CoV-2 infection was detected in the laboratory of the Silesian Park of Medical Technology Kardio-Med Silesia (Zabrze, Poland). Throughout this period, infection with SARS-CoV-2 virus was confirmed in 14,340 cases and 1832 results were inconclusive (Table 1).

**Figure 1:**
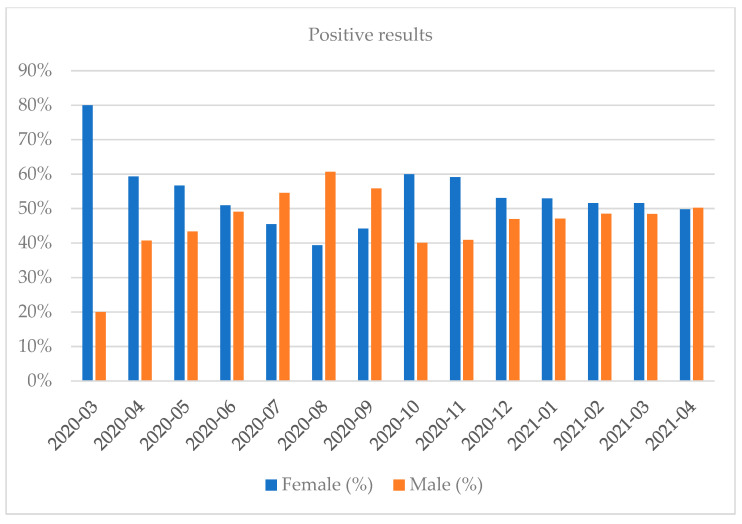
Distribution of positive results by gender in a given month.

**Figure 2:**
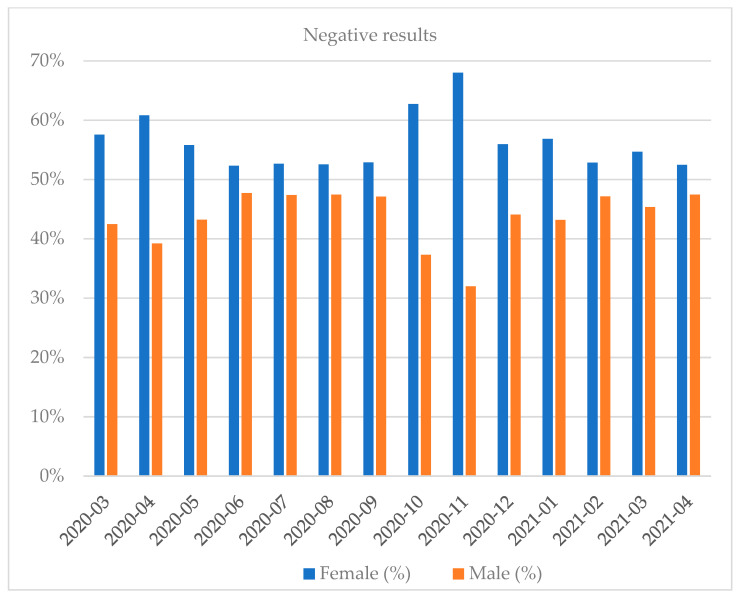
Distribution of negative results by gender in a given month.

**Figure 3:**
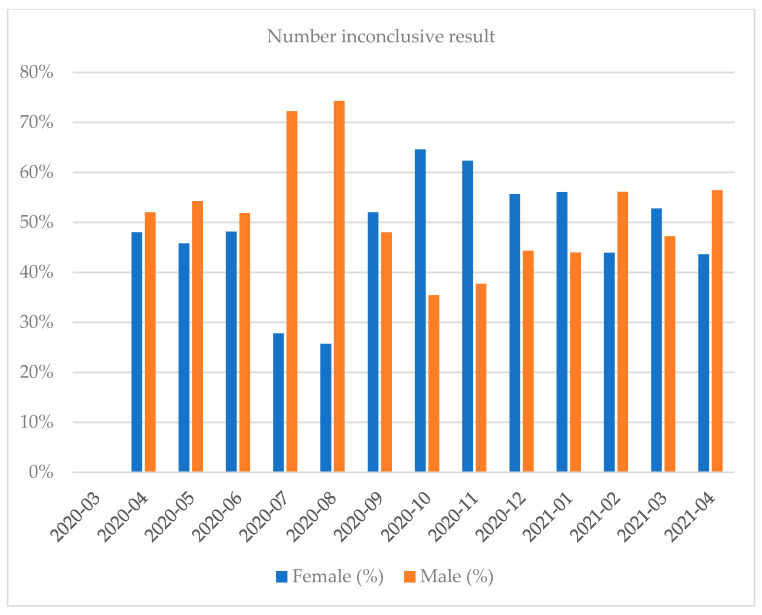
Distribution of inconclusive results by gender in a given month.

**Figure 4:**
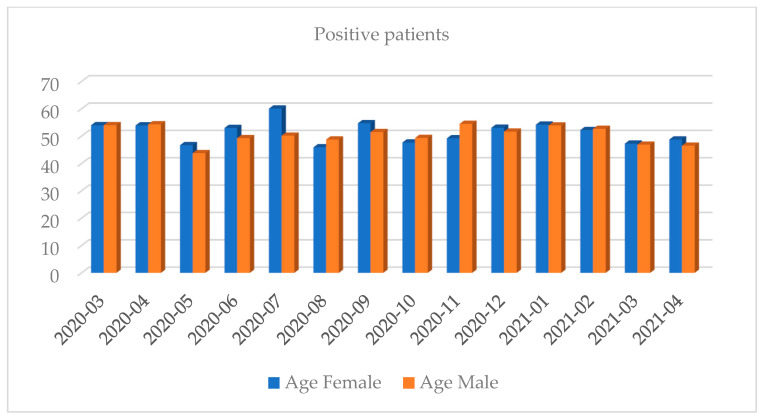
Distribution of positive results by the average age and gender in a given month.

**Figure 5:**
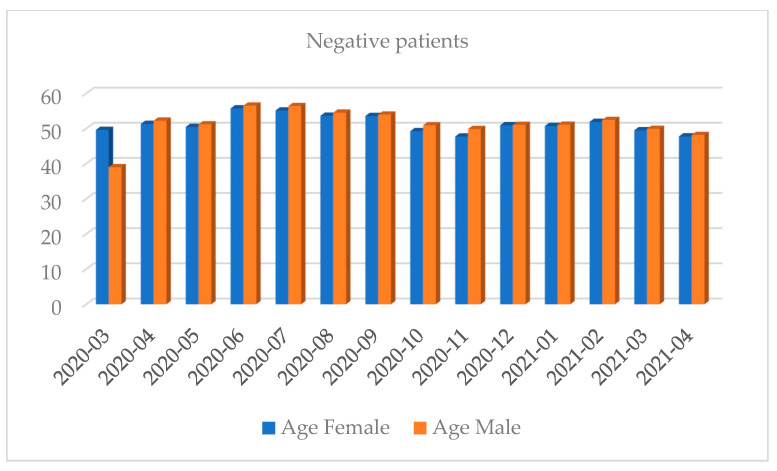
Distribution of negative results by the average age and gender in a given month.

**Figure 6:**
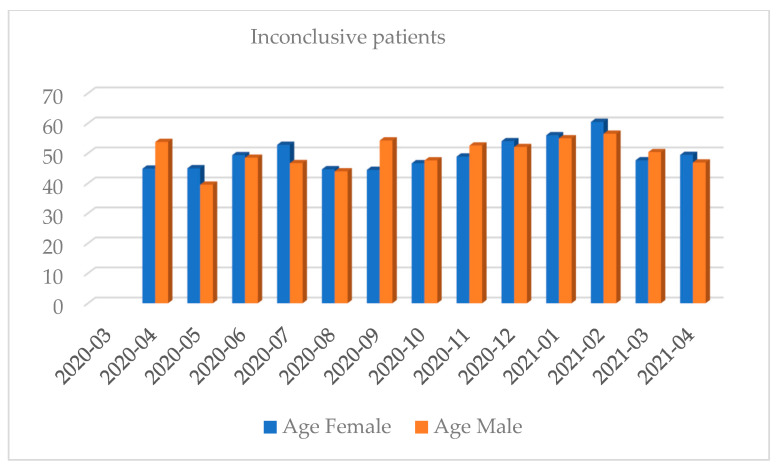
Distribution of inconclusive results by the average age and gender in a given month.

Women shared approximately 53.84% of the positive results total. Most of the tests were performed in October 2020 and the least in April 2020 (full months). Most of the positive results were recorded in October 2020 and the least in July 2020 (full months). The highest percentage of positive results regarding the share of all the results was recorded in March 2021 while the lowest was in July 2020 (full months) (Figure 1, Figure 2, Figure 3, Figure 4, Figure 5 and Figure 6).

Additionally, the authors reviewed a number of positive, negative or inconclusive results from the 15 largest cities of the Silesian region as compared to the 13-month period evaluated in our database. In October 2020 (the month showing the peak number of PCR tests) most of the positive, negative and inconclusive results came from Zabrze. The fewest positive results came from Jastrzębie-Zdrój, negative ones from Bielsko-Biała and the inconclusive ones from Mysłowice. In April 2020 (the month scoring the lowest record of PCR tests) most of the positive, negative and inconclusive results came from Bytom. The fewest positive and inconclusive results came from Gliwice and the negative ones from Jaworzno. Moreover, the urban population become infected with SARS-CoV-2 more often than the rural one as shown by the epidemiological analysis of infections in the 15 largest cities of the Silesian region.

Due to long-lasting symptoms in 422 patients, at least two RT-PCR tests were performed (Table 2). The mean age of these patients was 59.9 years and females shared 48% of the total cases. A detailed description is presented in Table 2.

The peak number of inconclusive results was recorded in October 2020 (353 cases) and the lowest one in July, 2020 (18 cases) (full month). A patient showing an inconclusive result was normally tested again within several dozens of hours. There were also cases of inconclusive results which followed a patient’s previous positive or negative tests. Based on the recorded data, we could distinguish among the following scenarios: (a) negative result → inconclusive result → positive result (3.95% of all inconclusive results); (b) positive result → inconclusive result → negative result (4.62% of all inconclusive results); (c) inconclusive result → positive result (16.04% of all inconclusive results); (d) inconclusive result → negative result (44.29% of all inconclusive results).

A total of 13 cases of reinfection with SARS-CoV-2 were detected. The average age of those patients was 57 years. Of that group, 4 patients (mean age = 73.4 years) were women and 9 were men (mean age = 51.8 years). The shortest interval between the first and the following infection was 60.25 days, the average time was 122.36 days, and the longest interval was 192.52 days.

Swab samples were collected from 11 patients potentially reinfected with SARS-CoV-2 (exception were 2 individuals from whom no samples were taken). In these patients the SARS-CoV-2 variants were identified (22 samples) using two reagent kits to ensure reproducible and reliable results. The results are presented in Table 3.

The presence of SARS-CoV-2 genetic material in samples from patients representing the first infection was confirmed only in seven cases, and in the stored samples from patients representing the second infection was confirmed only in 6 cases. The UK variant (lineages B.1.1.7) was confirmed in two patients (samples from April 2020 and 2021).

To verify the results obtained with GeneProof SARS-CoV-2 Vaccine Escape PCR Kit reactions were carried on the same samples using the VirellaSARS-CoV-2 Mutant Real Time RT-PCR Kit. The Wildtype of SARS-CoV-2 was confirmed in five samples representing the primary infection and three samples representing the secondary infection. In two samples representing the secondary infection no presence of SARS-CoV-2 RNA was confirmed. Four samples contained SARS-CoV-2-RNA Lineage B.1.258 or Cluster V (Mink, DK) (including two samples identified as the UK variant by the GeneProof SARS-CoV-2 Vaccine Escape PCR Kit as UK variant). SARS-CoV-2 nucleic acids were not detected in seven tested samples, probably due to degradation of SARS-CoV-2 viral RNA during long storage.

## 4. Discussion

As recommended by the WHO, potentially infected cases should be confirmed by amplification of the virus nucleic acid (NAAT) in RT-PCR tests. The samples should be collected from the respiratory tract: nasopharyngeal and oropharyngeal swab, sputum, endotracheal aspirate or bronchoalveolar lavage [9]. 

During the 13 months of our laboratory diagnostics for SARS-CoV-2 infections, the confirmed COVID-19 cases shared 13.21% of all RT-PCR test results. During that time, we carried out multiple testing for a number of medical and private organizations throughout the Upper Silesian region. Considering the perspective of the Silesian Voivodship worth mentioning is the study by Kowalska et al. who analyzed SARS-CoV-2 infections on the basis of data gathered by the Provincial Sanitary and Epidemiological Station in Katowice. Their results indicated that in early 2020, the infection rate reached approximately 5% in the Upper Silesian region [15].

On the other hand, Raciborski et al. analyzed the epidemiological situation in the first two months of the epidemic in Poland pointing to a higher notification rate for COVID-19 among women than in men with an average age of 50.6 years, contrary to our study where men shared 52% of the COVID-19 cases [16]. As for the age range, similar to our study, individuals over 50 were more frequently infected with COVID-19. Regarding the place of residence, our study confirmed that SARS-Co-2 infections were more common in the urban areas than in the rural environment [17,18].

Our study showed 1.69% of inconclusive RT-PCR test results. All of those were repeated to obtain a conclusive result, where over 44% produced a negative result. The initial inconclusive results might have resulted from excessive Ct cutoff and were common among the asymptomatic cases [19]. RT-PCR results are frequently affected by pre-analytical factors including viral inactivation procedures and transport buffers [20]. Having confirmed an inconclusive result our laboratory performed additional tests with the use of another RT-PCR kit (scarce) or another collection of a swab was recommended after 48 hours followed by a retesting procedure (prevalent).

Characterized by relatively long persistence in the human body, SARS-CoV-2 may prove a potential reason for positive RT-PCR test in recovery patients. There have been several reports of persistent viral shedding after the onset of COVID-19 symptoms, based on RT-PCR testing: 24 days [21] up to 6 weeks [22], over 45 days [23], 60 days [24], 92 days [25]. Moreover, the meta-analysis of 43 studies and 3229 cases revealed that the average duration of SARS-CoV-2 shedding was 17 days while the maximum was as long as 83 days [26]. Interestingly, longer viral shedding was observed in symptomatic patients rather than asymptomatic ones, especially among those who presented chest pain or sputum [21,23]. Our study pointed to the maximum interval between two positive results RT-PCR tests reaching 52.88 days, with the average above 29 days. In addition, in one case, we performed eight RT-PCR tests results with the interval between two positive results reaching 48 days, confirming the persistent viral shedding of SARS-CoV-2.

Reinfection with respiratory viruses may be associated with a weak immunological response or reinfection with another genotype [27]. The Centers of Disease Control and Prevention (CDC) presented criteria for detection of potential reinfection, as well as positive RT-PCR, in patients more than 90 days after the primary infection with or without any symptoms or for a positive RT-PCR result in a symptomatic case during days 45 through 89 since the first infection [28]. Genomic sequencing is required to differentiate between reinfection and the persistent viral shedding from the first infection [28] and the first cases of reinfection with COVID-19, confirmed by next-generation sequencing, were reported in patients in the USA. The positive results for the first and the second infection were separated by an interval of only 48 days [29] or 142 days after the first infection [30], 51 days [31], 82 days with virus from the same clade [32], and 3 months with virus from a different lineage [33]. Our study showed 13 cases of a positive RT-PCR result after a longer period of time following termination of the first confirmed infection of COVID-19, which we interpret as reinfection. A cutoff limit of 60 days was taken into consideration.

Interestingly, positive RT-PCR results have been observed after two consecutive negative results [34,35,36], which was explained by prolonged nucleic acid conversion and a false-negative result of the diagnostic method [37]. Prolonged nucleic acid conversion is defined as the time between the day of the symptoms onset and the day of the first negative result of a RT-PCR test [37] and during this time the results of RT-PCR testing may vary continuously during up to 70 days, which is important for the diagnosis of potential reinfection [38].

A study based on 619 cases confirmed that around 14% of patients returned to a positive RT-PCR result in a median time of 7 days (range 2–19 days) [35], while another study revealed a high percentage of negative RT-PCR test results [39]. Intriguingly, some patients showing manifestations of pneumonia as well as characteristic symptoms of SARS-CoV-2 required several RT-PCR tests to confirm the infection [40,41]. A systematic review reported rates of 0.018–0.033 for nasopharyngeal false-negative sample [42]. A false-negative result may be connected with lower RNA virus copy in a specimen [43], a window period (first 3–5 days) between the acquisition of infection and detectability [44], or thermal inactivation [45] or with the type of sample, such as a throat swab [22,46] which reduces sensitivity of RT-PCR [47]. In SARS-CoV-2 showing rapid evolution and genetic diversity [48], the combination of multiple assay panel in RT-PCR tests may decrease the risk of inconclusive or false results [49].

In an attempt to determine SARS-CoV-2 variants with the available samples of potentially reinfected patients, two samples showed discrepant results when CE IVD reagents were used, but although these reagents should be reliable the variants detected with one RT-PCR test (B.1.1.7, UK) were not confirmed by the second but were classified as B.1.258/Cluster V variant. This variant has been present in Central Europe since August 2020, and the Cluster V variant is the mink-related variant of SARS-CoV-2 discovered in North Jutland in November 2020 [50]. Brejová et al. examined samples collected from various regions of Slovakia at the end of 2020 which presumably contained variant B.1.1.7 due to the travelers’ routes or the history of contact with virus carriers [51], and by sequencing, variant B.1.1.7 was confirmed in some samples while others showed the presence of B.1.258 (circulating throughout Central Europe since August 2020). These results show that sequencing is the most reliable method to determine the variants of SARS-CoV-2. RT-PCR methods are inexpensive and suitable for large-scale screening, nevertheless, the detected variant of SARS-CoV-2 in a sample should be confirmed by sequencing [52], and therefore, in the future we plan to sequence the collected samples to confirm the detected SARS-CoV-2 variants.

This study has the limitation that we did not analyze all the examined patients from the entire region of Upper Silesia, and to obtain and analyze the results from other laboratories in the Silesian Voivodeship it would be necessary to obtain additional approvals.

## 5. Conclusions

Due to the higher risk of SARS-CoV-2 infection among the Upper Silesian population, the region is at greater risk of a deteriorating economic situation and healthcare, as compared to other areas of Poland. RT-PCR methods for the detection of SARS-CoV-2 variants are inexpensive and suitable for large-scale screening, but they can be untrustworthy and results should be confirmed by sequencing.

## Figures and Tables

**Table 1:** Number of tests per month and type of result.

Month and Year	Number of Tests	Positive Results, *n* (%)	Negative Results, *n* (%)	Number of Inconclusive Results, *n* (%)
2020-03	144	5 (3.47)	139 (96.53)	0 (0.00)
2020-04	4718	457 (9.69)	4236 (89.78)	25 (0.53)
2020-05	9050	256 (2.83)	8604 (95.07)	190 (2.10)
2020-06	8935	104 (1.16)	8804 (98.53)	27 (0.30)
2020-07	6989	33 (0.47)	6938 (99.27)	18 (0.26)
2020-08	7301	61 (0.84)	7205 (98.69)	35 (0.48)
2020-09	6354	77 (1.21)	6252 (98.39)	25 (0.39)
2020-10	13,792	3173 (23.01)	10,266 (74.43)	353 (2.56)
2020-11	12,896	2676 (20.75)	9936 (77.05)	284 (2.20)
2020-12	7324	1335 (18.23)	5777 (78.88)	212 (2.89)
2021-01	7042	907 (12.88)	5978 (84.89)	157 (2.23)
2021-02	6392	745 (11.66)	5565 (87.06)	82 (1.28)
2021-03	10,411	2723 (26.16)	7436 (71.42)	252 (2.42)
2021-04	7168	1788 (24.94)	5208 (72.66)	172 (2.40)
**Total**	108,516	14,340 (13.21)	92,344 (85.10)	1832 (1.69)

**Table 2:** Patients according to long-lasting symptoms.

Number of PCR Tests Performed	Number of Patients	Average Age (Years)	Female (%)	Minimal Interval between Two Extreme Positive Results (Days)	Average Interval between Two Extreme Positive Results (Days)	Maximum Interval between Two Extreme Positive Results (Days)
2	362	58.42 ± 14.82	85.78	1.99	16.87 ± 9.99	52.88
3	46	67.22 ± 11.69	56.52	7	21.98 ± 4.79	37.67
4	9	72.22 ± 12.37	33.33	19.22	24.73 ± 3.73	33.09
5	3	79.67 ± 1.78	33.33	25.96	29.42 ± 2.59	33.31
6	1	83.00 ± ns	100	-	35.41 ± ns	-
8	1	81.00 ± ns	100	-	48.71 ± ns	-

**Table 3:** Interpretation of results for the identified SARS-CoV-2 variants.

	First Infection		Second Infection	
Patients Number	GeneProof SARS-CoV-2 Vaccine Escape PCR Kit	VirellaSARS-CoV-2 Mutant Real Time RT-PCR Kit	GeneProof SARS-CoV-2 Vaccine Escape PCR Kit	VirellaSARS-CoV-2 Mutant Real Time RT-PCR Kit
1.	NR	NR	Wildtype	Wildtype
2.	Wildtype	Wildtype	NR	NR
3.	Wildtype	Wildtype	NR	NR
4.	Wildtype	B.1.258/Cluster V	UK	B.1.258/Cluster V
5.	UK	B.1.258/Cluster V	Wildtype	NR
6.	NR	NR	Wildtype	Wildtype
7.	Wildtype	Wildtype	NR	NR
8.	Wildtype	Wildtype	NR	NR
9.	NR	NR	Wildtype	NR
10.	Wildtype	B.1.258/Cluster V	NR	NR
11.	Wildtype	Wildtype	Wildtype	Wildtype

Abbreviations: NR, no result; B.1.258, lineages B.1.258 SARS-CoV-2; Cluster V, SARS-CoV-2 variants related to mink; UK, lineages B.1.1.7 SARS-CoV-2.

## Data Availability

The data used to support the findings of this research are available upon request.

## Supplementary Materials

The following are available online at https://www.mdpi.com/article/10.3390/diagnostics12010007/s1, Table S1: CT values recommended by the manufacturers to confirm a positive result for the presence of SARS-CoV-2 virus genetic material and the method of interpretation of the inconclusive results in accordance with the recommendations of the Polish National Institute of Hygiene.
