# Supplementary material for: RT-PCR Detection of SARS-CoV-2 among Individuals from the Upper Silesian Region—Analysis of 108,516 Tests
Source: Diagnostics (Basel). 2021 Dec 21;12(1):7. doi: 10.3390/diagnostics12010007 (PMC8774892; doi:10.3390/diagnostics12010007)
Supplement: Supplementary file 1 [file diagnostics-12-00007-s001.zip › diagnostics-1497884-supplementary.pdf]

*Article*

# RT-PCR detection of SARS-CoV-2 among individuals from the Upper Silesian region - analysis of 108 516 tests.

**Adam Konka, Mateusz Lejawa, Jadwiga Gaździcka, Aneta Bochenek, Martyna Fronczek and Joanna Katarzyna Strzelczyk**

**Table S1.** CT values recommended by manufacturers to confirm a positive result for the presence of SARS-CoV-2 virus genetic material and the method of interpretation of inconclusive results in accordance with the recommendations of the Polish National Institute of Hygiene.

| **Test name** | **Ct value for positive results** | | | | | **Interpretation of inconclusive results** |
|---|---|---|---|---|---|---|
| | **ORF1ab gene** | **N gene** | **E gene** | **RdRP Gene** | **IC*** | |
| **COVID-19 Real Time Multiplex RT-PCR Kit (Labsystems Diagnostics Oy, Vantaa, Finland)** | ≤ 40 | ≤ 40 | ≤ 40 | - | ≤ 35 or / | The results for test samples containing the amount of SARS-CoV-2 virus genetic material at the limit of analytical sensitivity ("weak positive result"), i.e. + -> 35 Ct (indicative value, depending on the PCR kit used), it is recommended to report as an inconclusive result and have another patient sample taken for testing 24-48 hours after collecting the sample for which the positive result was questionable. |
| **SARS-CoV-2 Real Time PCR LAB-KIT (BioMaxima S.A., Lublin, Poland)** | ≤ 38 | ≤ 38 | - | - | ≤ 38 | |
| **Bosphore Novel Coronavirus (2019-nCOV) Detection Kit (Anatolia Genework, Istanbul, Turkey)** | < 32 | - | < 32 | - | < 30 | |
| **DiaPlexQ Novel Coronavirus (2019-nCoV) Detection Kit (SolGent Co., Ltd., Daejeon, Korea)** | ≤ 40 | ≤ 40 | - | - | ≤ 26 | |
| **Liferiver Novel Coronavirus (2019-nCoV) Real Time Multiplex RT-** | ≤ 41 | ≤ 41 | ≤ 41 | - | / | |

| | | | | | |
|---|---|---|---|---|---|
| **PCR Kit (Shanghai ZJ Bio-Tech Co., Ltd., Schanghai, China)** | | | | | |
| **LightMix Modular SARS-CoV-2 (Covid19) RdRP, E-gene (Roche, Basel, Switzerland)** | - | - | < 36 | < 40 | / |
| **Novel Coronavirus (2019-nCoV) Nucleic Acid Diagnostic Kit (Sansure Biotech, Changsha, China)** | ≤ 40 | ≤ 40 | - | - | ≤ 40 |
| **VIASURE SARS-CoV-2 Real Time PCR Detection Kit (Certest Biotec S.L., Saragossa, Spain)** | < 38 | < 38 | - | - | / |

Abbreviations: "-" gene is not present in this kit; * IC (Internal Control) The presence of an internal control depends on the RT-PCR kit. Some kits have an Internal Control that determines the detection of a human gene in a sample. Detection of Internal Control (depending on the kit) is not required if there is a positive result in any of the other detection channels; "/" no requirement to analyze.
